# Supplementary material for: Effect of urban habitat use on parasitism in mammals: a meta-analysis
Source: Proc Biol Sci. 2020 May 13;287(1927):20200397. doi: 10.1098/rspb.2020.0397 (PMC7287365; doi:10.1098/rspb.2020.0397)
Supplement: Table of Studies [file rspb20200397supp1.pdf]

Sup 1. Table of studies included in meta-analysis, along with species and urban locations of the populations included in each study.

Study	Host species	Urban Location, Country
Hill et al. 2008 [60]	*Trichosurus vulpecula*	Sydney, Australia
Thompson et al. 2010 [61]	*Isoodon obesulus*	Perth, Australia
Paparini et al. 2012 [62]	*Rattus rattus*	Darwin City, Australia
Dowle et al. 2013 [63]	*Parameles nasuta*	Sydney, Australia
Hillman et al. 2017 [64]	*Isoodon obesulus*	Perth, Australia
Seethamchai et al. 2008 [65]	*Macaca fascicularis*	Prachuap Khiri Khan, Thailand
Lane et al. 2011 [41]	*Macaca fascicularis*	Denpasar, Indonesia
Debenham et al. 2017 [66]	*Macaca mulatta*	Chandrigarh and Shimla, India
Adrus et al. 2018 [67]	*Macaca fascicularis*	Langkawi, Perak State, and Selangor State, Malaysia
Kumar et al. 2018 [68]	*Macaca radiata*	Bangalore, Mettupalayam, and Ooty, India
Anders et al. 2019 [69]	*Apodemus speciosus*	Obihiro, Japan
Jeneby et al. 2002 [70]	*Cercopithecus mitis*	Thika, Kenya
Fagir & El Rayah 2009 [71]	*Arvicanthis niloticus*	Khartoum, Sudan
Jeneby et al. 2011 [72]	*Cercopithecus mitis*	Kibwezi, Kenya
Ravasi et al. 2012 [73]	*Papio ursinus*	Cape Town, South Africa
Froeschke & Matthee 2014 [74]	*Rhabdomys pumilio*	Stellenbosch, Somerset West, Franschoek University, and Khayelitsha, South Africa
Antolova et al. 2004 [75]	*Vulpes vulpes*	Slovak Republic
Hegglin et al. 2007 [76]	*Vulpes vulpes*	Zurich, Switzerland
Reperant et al. 2007 [77]	*Vulpes vulpes*	Geneva, Switzerland
Robardet et al. 2008 [78]	*Vulpes vulpes*	Nancy, France
Reperant et al. 2009 [79]	*Arvicola terrestris*	Geneva, Switzerland
Kataronovski et al. 2011 [80]	*Rattus norvegicus*	Belgrade, Serbia
Duscher et al. 2015 [81]	*Vulpes vulpes*	Vienna, Austria

Svitalkova et al. 2015 [82]	*Apodemus flavicollis; Myodes glreolus*	Bratislava, Slovakia
Dwuznik et al. 2017 [83]	*Apodemus agrarius; Apodemus flavicollis*	Warsaw, Poland
Kruecken et al. 2017 [84]	*Apodemus agrarius; Apodemus flavicollis*	Berlin, Germany
Tsakmakidis et al. 2017 [85]	*Mus musculus; Rattus rattus*	Thessaloniki, Chalkidiki, Kilkis and Imathia, Greece
Gryczynska et al. 2018 [86]	*Apodemus agrarius; Apodemus flavicollis*	Warsaw, Poland
Jacobson et al. 1982 [87]	*Procyon lotor*	West Lafayette, IN, United States
Gomez et al. 2008 [88]	*Peromyscus leucopus; Sciurus carolinensis*	Baltimore, MD and Washington D.C., United States
Blizzard et al. 2010 [89]	*Procyon lotor*	Clarke County, GA, United States
Lehrer et al. 2010 [43]	*Marmota monax*	Champaign-Urbana, IL, United States
Jardine et al. 2012 [90]	*Procyon lotor*	Niagara, Canada
Kellner et al. 2012 [91]	*Peromyscus spp.*	Chicago, IL, United States
Pipas et al. 2014 [92]	*Procyon lotor*	WY, United States
Watts et al. 2015 [42]	*Canis latrans*	Calgary, Canada
Lewis et al. 2017 [93]	*Lynx rufus; Puma concolor*	Boulder, CO, United States
Panti-May et al. 2017 [94]	*Mus musculus; Rattus rattus*	San José Tecoh, Mexico
Hartnett et al. 2018 [95]	*Procyon lotor*	Ontario, Canada
Alonso et al. 2005 [96]	*Alouatta guariba*	Porto Alegre, Brazil
Fornazari et al. 2011 [97]	*Didelphis albiventris*	Botucatu Municipality, São Paulo State, Brazil
Kowalewski et al. 2011 [98]	*Alouatta caraya*	Cerrito and San Cayetano, Argentina
Roque et al. 2012 [99]	*Philander opossum*	Abaetetuba, Brazil
Soto-Calderon et al. 2016 [100]	*Saguinus leucopus*	Medellín, Colombia
Fitte et al. 2017 [101]	*Rattus spp.*	Gran La Plata, Argentina
do Carmo-Silva et al. 2019 [102]	*Holochilus sciureus*	São Bento City, Brazil
