# Supplementary material for: Effect of urban habitat use on parasitism in mammals: a meta-analysis
Source: Proc Biol Sci. 2020 May 13;287(1927):20200397. doi: 10.1098/rspb.2020.0397 (PMC7287365; doi:10.1098/rspb.2020.0397)
Supplement: Funnel plot of log odds ratios [file rspb20200397supp2.pdf]

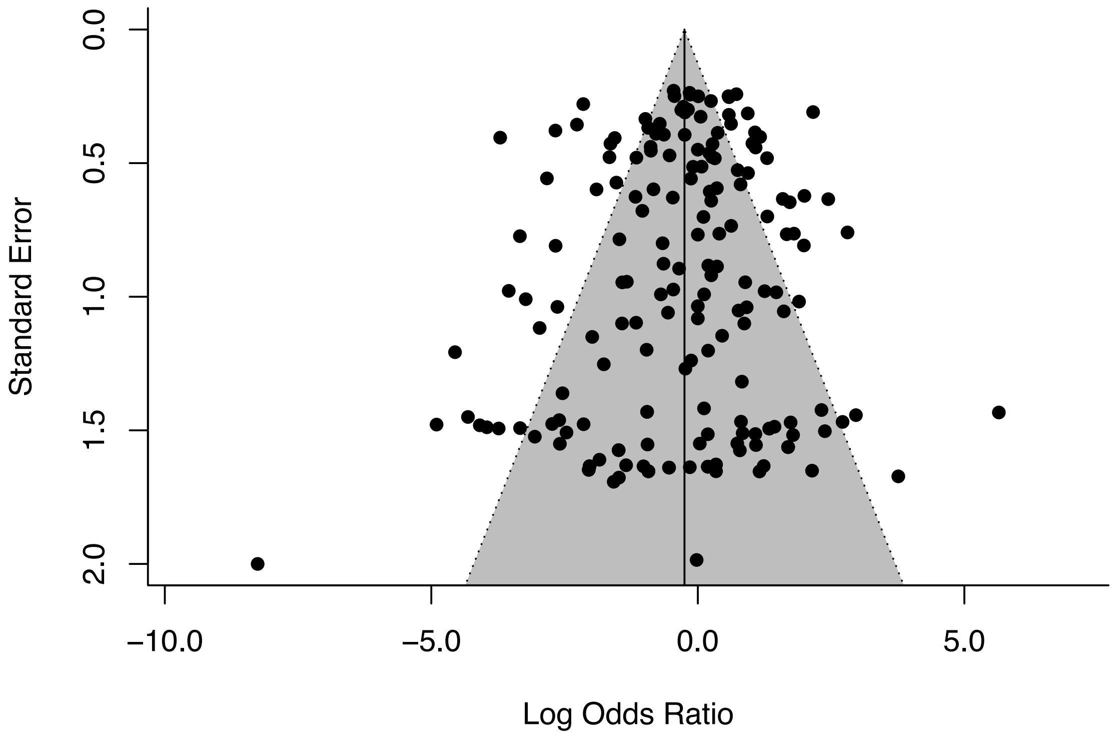

Sup 2. Funnel plot of the effect sizes, calculated as log odds ratios, plotted against the standard errors of the effect sizes. Each point represents a unique host-parasite combination. A test for funnel plot asymmetry was not significant ($z = -1.30$, $p=0.19$).
